# Current status of pharmacotherapy for primary sclerosing cholangitis

**DOI:** 10.3389/fmed.2025.1544601

**Published:** 2025-06-27

**Authors:** Hang Yang, Juan Zhen, Xiaoyan Huang, Minqi Chen, Hongsi Cui, Xia Sheng, Xinyu Li

**Affiliations:** ^1^Department of Anesthesiology, The First Hospital of Jilin University, Changchun, Jilin, China; ^2^Department of Cadre Ward, The First Hospital of Jilin University, Changchun, Jilin, China; ^3^The First Hospital of Jilin University, Changchun, Jilin, China; ^4^Department of Clinical Nutrition, China-Japan Union Hospital of Jilin University, Changchun, Jilin, China; ^5^Department of Intensive Care Medicine, The First Hospital of Jilin University, Changchun, Jilin, China

**Keywords:** primary sclerosing cholangitis, ursodeoxycholic acid, Nor-UDCA, clinical trial, drug therapy

## Abstract

Primary sclerosing cholangitis (PSC) represents a cholestatic disease hallmarked by persistent and progressive inflammation of the bile ducts. Despite its low incidence and unfavorable prognosis, there is no pharmacological therapy capable of altering the course of PSC, and liver transplantation is the only effective treatment. In the face of the landscape of PSC, pharmaceutical therapy encounters great challenges that demand expeditious resolution. However, at present, many drugs have been carried out to phase III clinical trials and are expected to be applied to the clinical treatment of PSC patients in the future. This review integrates relevant research findings from PubMed and Web of Science databases up to October 2024 over the past decade, excluding other liver diseases, such as fatty liver disease, viral hepatitis, and alcoholic liver disease. It covers the vast majority of drugs currently in clinical trials, and focus on the summary of hot research drugs, and summarizes the latest drug-based therapeutic for PSC. This review not only provides certain information for clinical research and treatment of PSC, but it is also the first time that stem cell therapy has been linked to PSC, which is expected to improve cholestasis and liver inflammation in patients with PSC. The article provides explanations and comparisons of different drugs, offering a basis for future researchers to choose medications.

## 1 Introduction

Primary sclerosing cholangitis (PSC) is characterized by inflammatory destruction of intrahepatic or extrahepatic bile ducts, leading to bile stasis, fibrosis, and ultimately cirrhosis. According to the latest epidemiological data of European PSC clinical practice guidelines in 2022, the prevalence and incidence of PSC in Northern Europe are about 1/10,000 and 1/1,000,000, respectively (1). PSC is a progressive disease which is most common in males aged 30–40. Currently, there are no effective drugs to prevent the progression of PSC (2), and liver transplantation is the only effective treatment, with a 5-year survival rate of 85% after transplantation. The pathophysiological mechanisms of PSC have not yet been fully elucidated, but it is currently believed to be the result of the interplay of multiple factors, including immune factors, genetic factors, bile acid metabolism disorders, and gut microbiota dysbiosis. The gut microbiome is altered (i.e., dysbiosis) in patients with PSC and is characterized by intestinal mucosal inflammation and impaired intestinal barrier function (“leaky-gut” phenomenon). These alterations may allow intestinal microbes and/or their metabolites or antigens to enter the circulation and activate the immune response in the liver. Aberrant migration of intestinal lymphocytes and translocation of microbial products or metabolites, which may activate innate and adaptive immune responses in the liver. This immune activation further leads to the damage and inflammatory reaction of cholangiocytes. Cholangiocytes respond to immune activation leading to cholestasis which further exacerbates inflammatory responses and cellular senescence. Cholangiocyte injury and cholestasis are key links in the pathophysiology of PSC, which interact with each other in a vicious cycle. The periductular glands hyperplasia, the periductular mesenchymal cells acquire myofibroblast phenotype, leading to large bile duct fibrosis. These changes eventually lead to bile duct stricture and hepatic fibrosis, which in turn may progress to cirrhosis and liver failure. These pathophysiologic mechanisms provide potential targets for the treatment of PSC, such as modulation of gut microbiota, suppression of immune activation, improvement of cholangiocyte function and anti-fibrotic therapy (3–5). There is an urgent need to develop effective drugs to improve the clinical outcomes of PSC patients. The current therapeutic mainly focus on alleviating symptoms and resolving complications, including fever, jaundice, pruritus, portal hypertension, ascites, and so on, but cannot fundamentally delay or reverse the progression of the disease, which brings limitations and challenges to clinical management. At present, pharmaceutical interventions for PSC remain in the clinical trial stage, demonstrating promising preliminary prospects, ursodeoxycholic acid (UDCA), among the investigated compounds, is the most widely studied and commonly employed drug in clinical, many emerging pharmaceutical agents is advancing through the developmental pipeline. Therefore, this article summarizes the current researches in pharmaceutical therapy of PSC in 10 years (Tables 1, 2), in order to provide insights for effective treatment of PSC.

**TABLE 1:** Summary of new PSC therapeutic drugs currently in phase II clinical practice.

Drugs	Mechanism	Clinical trial	Research status
Nor-UDCA	Side chain shortening derivatives of UDCA	Phase II (NCT01755507)	The study confirmed the efficacy and safety of oral three doses of Nor-UDCA (500, 1,000, or 1,500 mg/d) and placebo in PSC patients. Conclusion: Nor-UDCA can reduce ALP levels and show good safety.
OCA	FXR agonist	Phase II (NCT02177136)	In this study, patients were randomly divided into 1:1:1, and received placebo, OCA 1.5–3.0 or 5–10 mg once a day for 24 weeks. Conclusion: OCA 5–10 mg treatment can reduce serum ALP in patients with PSC.
Cilofexor	FXR agonist	Phase II (NCT02943460)	The study lasted 12 weeks and was randomly assigned to Cilofexor 100 mg, 30 mg, and placebo. The results showed that all patients had changes in liver biochemistry and serum fibrosis markers.
NGM282 (M70)	FGF19 analogs	Phase II (NCT02704364)	In a 12-week study, 62 subjects were divided into three groups and received NGM282 1 mg, 3 mg, or placebo, respectively. The results showed that NGM282 could effectively inhibit bile acid synthesis and reduce fibrosis markers, but had no significant effect on ALP level.
CS0159	FXR agonist	Phase II (NCT05896137)	This trial studies the safety, tolerability, and efficacy of CS0159 tablets in the treatment of PSC patients, but the trial has not yet been completed.
Elafibranor	PPARα/β agonist	Phase II (EUCTR2022-002695-37-ES)	To evaluate the efficacy and safety of GFT505 in adult PSC patients, the trial is under recruitment.
Seladelpar	PPARδ agonist	Phase II (NCT04024813)	The study evaluated its efficacy and safety in PSC. The endpoint of the study was ALP level, and the results have not been published.
Volixibat	ASBT inhibitor	Phase II (NCT04663308)	A 28-week study is being recruited to evaluate its efficacy and safety in the treatment of pruritus caused by cholestasis in PSC patients.
A3907	ASBT inhibitor	Phase II (NCT05642468)	To evaluate the efficacy and safety of A3907, trials are underway to recruit.
LUM001	ASBT inhibitor	Phase II (NCT02061540)	This study shows that LUM001 can reduce the level of bile acid in PSC patients and significantly improve pruritus.
Vancomycin	Antibiotic	Phase II (NCT03710122)	The study assessed ALP levels and the rate of progression of liver fibrosis, but the results were not returned.
Vidofludimus calcium	Immunosuppressant	Phase II (NCT03722576)	The trial evaluated its safety and efficacy in PSC patients. The results showed that 27.7% of patients had normal ALP at week 24, well tolerated, and no drug-related serious adverse events.
CVC	Dual inhibitors of CCR2 and CCR5	Phase II (NCT02653625)	The test confirmed its safety and efficacy. The results showed that there was a certain degree of ALP reduction in PSC patients. The most common symptoms were rash, fatigue and dizziness, and were well tolerated.
CM-101	CCL24 monoclonal antibody	Phase II (NCT04595825) Phase II (EUCTR2018-004258-77-GB)	In order to evaluate its safety and efficacy in PSC patients, the trial has not been completed, and no relevant results have been reported. The aim is to study its safety and efficacy in PSC patients, but the trial has not yet begun.
Bexotegrast	αvβ6 integrin inhibitor	Phase IIa (NCT04480840)	To assess its safety and efficacy in PSC patients, a 12–48-week trial is being recruited and the results have not yet been returned.
HK-66oS	NAD+ enhancer	Phase II (KCT0006590)	To evaluate its safety and efficacy in PSC patients, a 12-week trial is being recruited and the results have not yet been returned.
HTD1801	The ionic salt of berberine + UDCA	Phase II (NCT0333928)	An 18-week study confirmed that HTD1801 can significantly improve ALP levels.

**TABLE 2:** Summary of new PSC therapeutic drugs currently in phase III clinical practice.

Drugs	Mechanism	Clinical trial	Research status
Nor-UDCA	Side chain shortening derivatives of UDCA	Phase III (NCT03872921)	It is expected to recruit 300 PSC patients to compare the therapeutic effects of Nor-UDCA (1,500 mg/d) and placebo on PSC. The study has not yet been completed.
Cilofexor	FXR agonist	Phase III (NCT03890120)	This study investigated the safety, tolerability and efficacy of Cilofexor in non-cirrhotic adult PSC patients, but has not yet been completed.
(UDCA+) bezafibrate	PPAR agonist	Phase III (NCT04309773)	This study evaluated the efficacy and safety of bezafibrate for 24 months in PSC patients who had received UDCA but still had cholestasis. The study has not yet been completed.
(UDCA+) fenofibrate	PPAR agonist	Phase III (IRCT2020042704722)	This study included 30 patients with PSC who were treated with UDCA before the study. The experimental group was given 200 mg fenofibrate and the control group was given placebo once a day for 6 months. The results showed that ALP was significantly reduced and the prognosis of PSC patients was better improved.
Vancomycin	Antibiotic	Phase III (NCT01802073)	The purpose of this study was to investigate the antibacterial and immunomodulatory effects of vancomycin in PSC patients with inflammatory bowel disease, but the results have not been published.

## 2 Materials and methods

The pharmaceutical information and research progress mentioned in the article are derived from relevant literature published in PubMed and Web of Science in the past 10 years up to October 2024, excluding other liver diseases, such as fatty liver disease, viral hepatitis, and alcoholic liver disease. We also conducted searches on International Clinical Trials Registry Platform to identify registered clinical trials, and then summarized and organized the data obtained from these sources.

## 3 Bile composition modulators

### 3.1 Ursodeoxycholic acid

Ursodeoxycholic acid is a naturally occurring bile acid that with nuclear receptors constituting its mechanism of action. The mechanisms of action for UDCA encompass the following: (1) stimulation of hepatocellular secretion; (2) promotion of cholangiocellular secretion; (3) exertion of anti-apoptotic effects; and (4) attenuation of bile toxicity (6). UDCA is considered as a primary therapeutic agent for the treatment of primary biliary cholangitis (PBC), however, its application in PSC remains contentious. The 2022 new European Association for the Study of the Liver (EASL) guidelines recommend a therapeutic dose range of 15–20 mg⋅kg^–1^⋅d^–1^ for UDCA in PSC, while the use of high-dose UDCA (defined as 28–30 mg⋅kg^–1^⋅d^–1^ in the guidelines) is strongly discouraged (1). Studies indicate that low-dose UDCA (13–15 mg/kg/d) can ameliorate liver biochemical indicators without no impacting on survival (7). Moderate dose (17–23 mg/kg/d) exhibit the potential to improve liver prognosis and survival trend (8). A randomized, double-blind, placebo-controlled trial in North America included 150 PSC patients, of which 76 patients receiving UDCA and 74 patients receiving placebo. The study was terminated prematurely by the sixth year due to the doubling risk of disease progression caused by UDCA (28–30 mg/kg/d). The study endpoints encompassed cirrhosis, varicose veins, cholangiocarcinoma, transplantation, or death (9). In addition, a cohort study confirmed that high-dose UDCA is associated with an increased risk of colorectal tumors in patients with ulcerative colitis, providing further evidence for the toxicity of UDCA within this weight-based dose range (10). A meta-analysis involving eight randomized clinical trials, exploring varied UDCA doses ranging from 13–15 to 28–30 mg/kg/d, compared UDCA against placebo or no intervention. These trials demonstrated that UDCA within this dose range did not impede the progression of PSC (11). However, the prevalent use of low to moderate UDCA doses in PSC suggests the necessity for further prospective research. Moreover, UDCA as primary treatment, it is used in combination with peroxisome proliferator-activated receptor (PPAR) agonists, or antibiotics will be introduced in subsequent sections.

### 3.2 24-Norursodeoxycholic acid

24-Norursodeoxycholic acid (Nor-UDCA) is a side chain shortening derivative of UDCA inherent resistance to side chain conjugated. In contrast to UDCA, which partakes in a comprehensive enterohepatic circulation between the intestine and liver, Nor-UDCA engages in biliary hepatic shunt between bile duct cells and liver cells, producing high cholesterol rich in bicarbonate (HCO_3_^−^) and reducing the toxicity of bile to epithelial structures. The biliary hepatic shunt not only serves this protective function but also contributes to the accumulation of drugs within the liver. Besides its beneficial effects on the biliary system, Nor-UDCA also exhibits a spectrum of therapeutic functions, including anti-proliferative, pro-regenerative, anti-inflammatory, and anti-fibrotic functions, thereby conferring direct liver protective properties (12). In a 4-week experiment employing Mdr2 (−/−) mouse model of primary sclerosing cholangitis (PSC), it was found that Nor-UDCA can improve the sclerosing cholangitis in mice (13). A randomized controlled trial (NCT01755507) involving 38 centers from 12 European countries evaluated the efficacy and safety of three oral doses of Nor-UDCA (500, 1,000, or 1,500 mg/d) in comparison to a placebo among PSC patients. The results indicated a notable reduction in alkaline phosphatase (ALP) levels and underscored the overall safety of Nor-UDCA (14). The study is of high quality as a randomized controlled trial with low risk of bias. However, due to the small sample size and short follow-up duration, its results need to be further validated in larger-scale and longer-term clinical trials. A large randomized, double-blind, placebo-controlled phase III clinical trial (NCT03872921) is currently underway, with an estimated recruitment of 300 PSC patients, this trial seeks to discern the therapeutic effects of Nor-UDCA administered at a dosage of 1,500 mg/d versus a placebo, further contributing to our understanding of Nor-UDCA’s potential as a treatment modality for PSC.

### 3.3 FXR agonists and downstream target molecules

The farnesol receptor (FXR), a bile acid activated nuclear receptor mainly expressed in the liver and intestines, plays an outstanding role in regulating genes associated with metabolic processes of bile acid synthesis, transport, and reabsorption, and participates in carbohydrate and lipid metabolism. Due to its centrality in these crucial physiological processes, FXR is recognized as a promising pharmacological target for the treatment of liver diseases associated with bile acid dysregulation. Its mechanism of action consists of controlling the expression of bile salt export pump (BSEP) on the membrane of hepatocytes bile canaliculi, as well as the expression of organic solute transporter (OST) α and β in the intestine and distal part of the bile duct. In addition, FXR induces the expression of small heterodimer partner (also known as corepressor superfamily 0) (15). Obeticholic acid (OCA), a semi-synthetic derivative of chenodeoxycholic acid (CDCA), has been identified as an effective ligand for FXR. In a phase II randomized, double-blind, placebo-controlled, dose-seeking study, patients were randomly assigned in a ratio of 1:1:1 and received either a placebo, OCA at doses ranging from 1.5 to 3.0 mg, or OCA at doses spanning 5 to 10 mg once daily over a 24-week treatment period. The results showed that the administration of OCA at the higher dosage range of 5–10 mg can reduce serum ALP in PSC patients. Furthermore, it is noteworthy that mild to moderate dose-dependent pruritus emerged as the most frequently observed adverse event during the course of the treatment, underscoring the need for careful consideration of dosage in therapeutic strategies involving OCA for PSC patients(16) (NCT02177136).

Besides OCA, Cilofexor (GS-9674), a non-steroidal FXR agonist, is under investigation for use in the treatment of PSC. In a phase II double-blind, placebo-controlled study, the efficacy and safety of Cilofexor were tested in PSC patients. The patients were randomly assigned to receive Cilofexor at doses of 100 mg (*n* = 22), 30 mg (*n* = 20), or placebo (*n* = 10) orally once a day for 12 weeks. The results demonstrated notable alterations in liver biochemistry and serum fibrosis markers across all treated patients (NCT02943460). Subsequent analyses validated the favorable tolerability of Cilofexor alongside significant improvements in liver biochemical parameters and cholestasis indicators among PSC patients. For example, serum ALP, GGT, ALT, and AST were significantly reduced at week 96, but rebounded 4 weeks after treatment withdrawal. Cilofexor significantly decreased serum C4 (an intermediate of BA synthesis) and total BA levels, with an increase in FGF19 levels, indicating FXR activation and BA synthesis inhibition (17). Currently, an international randomized, double-blind, placebo-controlled phase III study is ongoing to investigate the safety, tolerability, and efficacy of Cilofexor in non-cirrhotic adult PSC patients (NCT03890120). Unfortunately, as of now, there is no data available for this study. But in a phase IIb clinical trial (NCT03449446) investigating its potential therapeutic effects on NASH related liver fibrosis, 198 patients with bridging fibrosis or compensatory cirrhosis were randomly divided into placebo, Cilofexor, Firsocostat (acetyl CoA carboxylase inhibitor), and a combination of Cilofexor and Firsocostat groups. The results showed that the combination of Cilofexor and Firsocostat treatment led to a significant decrease in the machine-read NASH CRN liver fibrosis score. Notably, the liver biopsy histology shifted from F3-F4 to ≤F2 phase liver fibrosis mode, indicating the specific therapeutic activity combination of Cilofexor and Firsocostat compared to single-drug regimens. It points out the promising therapeutic potential for the combination of Cilofexor and Firsocostat in advanced liver fibrosis related to NASH. Anticipation remains high for its implications in the context of PSC, however, whether it can prevent or even reverse the development of cirrhosis needs further research.

Fibroblast growth factor 19 (FGF19) is a component of the gut liver axis in maintaining bile acid homeostasis. It is induced by FXR activation and then reaches the liver to inhibit bile acid synthesis. Aldafermin, also known as NGM282 (M70), is a FGF19 analog that effectively inhibits CYP7A1, which encode the first rate-limiting enzyme in bile acid synthesis (18). In a double-blind, placebo-controlled phase II trial, 62 PSC patients exhibiting an increase in ALP (>1.5 × normal upper limit) were randomly assigned to receive NGM282 at doses of 1 mg, 3 mg, or placebo in a 1:1:1 ratio, administered once daily over a span of 12 weeks. The primary study endpoint focused on the alteration in ALP levels from baseline to week 12, while secondary study endpoint included changes in serum biomarkers of bile acid metabolism and fibrosis. Results at the 12-week unveiled no significant difference in the average change in ALP from baseline between the NGM282 group and the placebo group. Nevertheless, in comparison with the placebo group, NGM282 treatment exhibited significant enhancements in fibrosis biochemical markers, including liver fibrosis score. Consequently, this report concluded that the NGM282 can effectively inhibit bile acid synthesis and reduce fibrosis markers. However, it did not manifest a discernible impact on ALP levels in PSC (19) (NCT02704364).

All trans retinoic acid (ATRA) is an activator of FXR, effectively inhibit the synthesis of bile acids. A phase I clinical trial (NCT01456468) investigated the efficacy of moderate dose UDCA combined with ATRA (45 mg/m^–2^/d) in the treatment of PSC (20). The inclusion criteria were confirmed PSC for more than 6 months with prior inefficacy in responding to UDCA treatment alone. ATRA at 45 mg⋅d^–2^ and UDCA at 15–23 mg⋅d^–2^ were administered for 12 weeks to observe whether the serum ALP value decreased by 30% compared to pre-treatment levels. The results showed that after UDCA combined with ATRA treatment, the serum ALT and complement C4 levels in patients decreased significantly, but the primary observation quota ALP levels did not significantly decrease.

CS0159 is a novel potent non-steroidal FXR small molecule agonist developed through crystal structure-assisted design. Clinical trial approvals have been obtained for three indications, including NASH, PSC, and PBC. Notably, a phase I randomized, double-blind, placebo-controlled, single-dose incremental/multi-dose incremental study for PSC has been completed (NCT05082779); however, there is no relevant literature on this topic. Furthermore, another multicenter, randomized, 12-week, double-blind, placebo-controlled, and 40-week open phase II clinical trial is ongoing to evaluate the safety, tolerability, and efficacy of CS0159 tablets in patients diagnosed with PSC (NCT05896137).

In conclusion, FXR agonists and downstream target molecules, including OCA, Cilofexor, FGF19, and ATRA, are currently the focus of research, and some of them can improve biochemical indicators or fibrosis markers in patients with PSC, but the clear effect of treating PSC is still inconclusive. Although UDCA is currently a first-line drug, it is more suitable for patients with significant bile acid regulatory dysfunction. With the advancement of technology, the emergence of new FXR agonists or FGF19-related target molecules in the future cannot be ruled out, which can bring new hope for PSC treatment.

## 4 PPAR agonist

Peroxisome proliferator-activated receptor constitutes a family of nuclear receptors, with PPARα highly expressed in liver, heart and skeletal muscle, brown adipose tissue and kidney. In addition to its ability to regulate fatty acid metabolism, PPARα exhibits anti-inflammatory properties. Activation of PPAR can be achieved through the administration of antilipemic agents, such as bezafibrate. In a study from Japan, seven patients with PSC were subjected to oral administration of bezafibrate (400 mg/d), with subsequent analysis of its effect on ALT, ALP, AST, and GGT after 6 months. The results showed that liver enzyme levels of three patients were decreased, with the average ALP reduced to about 40% of the baseline (21). A joint French–Spanish study evaluated the efficacy and safety of fibrates in PSC patients. The investigation focused on the PSC patients who received fibrates (fenofibrate 200 mg/d or bezafibrate 400 mg/d) in conjunction with UDCA for 6 months after an incomplete biochemical response to treatment with UDCA (ALP ≥ 1.5 × normal upper limit). The results demonstrated that a noteworthy 41% reduction in ALP levels and significantly reduction in pruritus. This study suggests that the combination of UDCA and fibrates can improve biochemical indicators and pruritus in PSC patients with an incomplete response to UDCA, thereby providing a basis for a large-scale prospective study (22).

A retrospective observational study was conducted to assess the biochemical response and bile acid concentration in PSC patients exhibiting insufficient biochemical response under UDCA monotherapy in the combination of fenofibrate and UDCA. The results found that fenofibrate treatment resulted in a significant decrease in AST and ALT in PSC patients (23). In a subsequent randomized, double-blind, placebo-controlled phase III trial, 30 PSC patients (19 females and 11 males) previously treated with UDCA were enrolled. The experimental group received 200 mg of fenofibrate, and the control group received placebo once a day for 6 months. The results showed that ALP level was significantly reduced in the fenofibrate group, thereby contributing to improve the prognosis of PSC patients (24). Presently, a double-blind, multicenter, randomized, placebo-controlled phase III trial is underway to evaluate the efficacy and safety of 24-month treatment with fenofibrate in PSC patients who have received UDCA but continue to exhibit cholestasis (NCT04309773). This ongoing trial holds promise for advancing our understanding of fenofibrate’s role in managing PSC, particularly in cases where cholestasis persists despite UDCA intervention.

Elafibranor (GFT505) is a kind of PPARα/β agonist. It have been demonstrated that GFT505 can significantly reduce steatosis and hepatic inflammation and prevent the progression of fibrosis in the APOE*3-leiden CETP mouse model (25). A phase II clinical trial has demonstrated the safety and tolerability of GFT505, revealing a substantial reduction in ALP and bilirubin levels in PBC patients who demonstrated an incomplete response to UDCA (NCT03124108) (26). A phase II, multicenter, double-blind, randomized, placebo-controlled trial is currently recruiting to evaluate the efficacy and safety of GFT505 in adult patients with PSC (EUCTR2022-002695-37-ES). A phase II randomized, placebo-controlled trial was used to confirm its safety and efficacy in adolescent and pediatric patients with non-alcoholic hepatitis. However, the trial was terminated due to the lack of efficacy of GFT505 in adult fibrosis (NCT03551522). The future of GFT505 in the treatment of PSC, potentially with a focus on non-fibrotic adults, awaits further elucidation through rigorous scientific investigation and clinical exploration.

Seladelpar (MBX-8025) functions as a selective PPARδ agonist which can improve atherosclerosis and dyslipidemia. Notably, several researches indicate that MBX-8025 is capable to enhance insulin sensitivity, reverse dyslipidemia, mitigate hepatic accumulation of lipotoxic lipids, and ameliorate NASH pathology in diet-fed atherosclerotic obese diabetic mice in a mouse model of obesity, dyslipidemia and diabetes (27). A completed 24-week phase II, randomized, double-blind, placebo-controlled, multicenter clinical trial was engaged to evaluated the efficacy, tolerance and safety of MBX-8025 in PSC patients (NCT04024813). The primary endpoint of this trial centered on evaluating serum level of serum ALP in PSC patients post-24 weeks of treatment. However, the result has not been published yet. These outcomes are anticipated to provide substantial insights and support for further investigations and potential avenues for the continued study and exploration of MBX-8025.

In addition to bezafibrate and fenofibrate can reduce the level of ALP in PSC patients, the study of other PPAR agonists is still theoretical, the efficacy and safety are not clear, and further exploration is needed. Patients with PSC and hyperlipidemia may thus benefit more.

## 5 ASBT inhibitors

The apical sodium-dependent bile acid transporter (ASBT) located at the end of the ileum is a key transporter responsible for the intestinal reabsorption of bile acids, which is important for maintaining organic metabolic homeostasis. In recent years, ASBT inhibitors, targeting to inhibit enterohepatic recycling and change the bile acid recycling pool, have attracted increasing attention as specific drug with certain therapeutic potentials (28). Prominent ASBT inhibitors utilized in the management of cholestasis encompass lopixabat (A4250), odevixibat (SC-435), marilixibat (LUM001), GSK2330672, and so on. A randomized, double-blind, placebo-controlled phase II trial is presently underway to assess the efficacy and safety of Volixibat (LUM002) for the treatment of pruritus caused by cholestasis in PSC patients (NCT04663308). Additionally, another phase II trial evaluating the safety and efficacy, and tolerability of LUM001 in the treatment of PSC has been concluded (NCT02061540). The results showed that LUM001 exhibited a capacity to reduce the bile acid level in PSC patients and significantly improved pruritus symptoms (29). Furthermore, A3907, a selective ASBT inhibitor, was previously shown to be effective in removing bile acids and protecting the liver in mouse models, thereby providing a potential for further clinical development (30). A phase II trial is currently recruiting to evaluate the safety, efficacy and pharmacokinetics of A3907 in PSC patients (NCT05642468). This endeavor holds promise for advancing our understanding of A3907’s clinical utility in the context of PSC. Hopefully, this can bring hope to patients with impaired bile acid circulation.

However, there are few studies on other ASBT inhibitors, including A4250, SC-435, and GSK2330672, which may provide certain directions for future research.

## 6 Antibiotics

In recent years, many studies have found that recurrent biliary tract infections and intestinal microecological imbalance play an important role in the occurrence and development of PSC.

The pivotal role of the microbiome in chronic cholangiopathy, particularly in PSC, is acknowledged as a determinant influencing disease progression. The immune gut-liver axis in PSC may be fundamentally regulated through the intestinal microbiome. Antibiotics are commonly employed to modulate the intestinal microflora, exert their influence by reducing the gut bacterial load, thereby inhibiting the increase in the number of neutrophils in the liver. In light of the pathogenesis based on intestinal microbes, it is suggested that antibiotic treatment may delay the progression of chronic liver disease. Notably, metronidazole and vancomycin stand out as extensively studied antibiotics in this context. Metronidazole manifests beneficial effects on intestinal inflammation. It exhibits the capacity to decrease colonic permeability of bacterial endotoxin, inhibit endotoxin-induced TNF-α production, suppress the secretion of chemokine and cytokine by biliary epithelial cells, ultimately resulting in the attenuation of hepatic inflammation. In a randomized controlled trial involving 80 adult PSC patients, with a focus on microbiome modulation, subjects were randomized to receive UDCA at a dose of 15 mg/kg/d in combination with metronidazole or UDCA alone for a duration of 36 months. The results revealed a significant reduction in ALP, as well as improvements in histologic staging and grading within the group receiving the combined metronidazole and UDCA regimen (31). This underscores the potential therapeutic efficacy of targeted microbiome modulation in the management of PSC.

Cytokines play an important role in the inflammatory cascade events. TNF-α is a cytokine produced by monocytes/macrophages. The release of TNF-α can be stimulated by Gram-negative bacterial products such as lipopolysaccharide, and the cell walls of Gram-positive bacteria. Antibiotics exert their influence by diminishing the exposure of the biliary epithelium to pathogens, a process involving the reduction of endotoxin. Vancomycin, among these antibiotics, has been observed to down-regulate TNF-α in the bloodstream. However, oral vancomycin is difficult to absorb from the gastrointestinal tract, suggesting that the response of vancomycin may be due to its antimicrobial effect on unknown pathogens or normal flora that cause abnormal immune responses in the liver. Two early clinical trials published in 2013 and 2016 explored the effect of oral vancomycin on PSC. In a Mayo study, 35 adult PSC patients were randomly assigned to receive either two doses of vancomycin (125 mg or 250 mg, 4 times per day) or metronidazole (250 mg or 500 mg, 3 times per day). After 12 weeks, a statistically significant reduction in serum ALP in the vancomycin group was observed, whereas no significant difference was noted in the metronidazole-treated group (32). In another randomized placebo-controlled trial, after 12 weeks of UDCA treatment, 11 patients received a placebo, while 18 were administered vancomycin of 125 mg four times daily. A statistically significant reduction in ALP was found in the vancomycin group. No statistically significant difference was observed in the placebo group (33). The efficacy of vancomycin constrained by its temporal impact during the course of treatment, as evidenced by the resurgence of liver biochemical irregularities within a few weeks following treatment discontinuation (34). On the contrary, protracted or continuous administration of antibiotics will cause a variety of side effects, with a heightened risk of infections with multi-drug resistant bacteria (35). A recent phase III study of vancomycin (NCT01802073) was completed. However, data suggested that only 9/14 and 9/20 adults were able to complete the study. Furthermore, another multicenter phase II clinical trial (NCT03710122) is underway, employing randomization of participants to receive either a placebo or vancomycin. This trial aims to assess whether serum ALP values will be normal at 6, 12, and 18 months of treatment. Additionally, the assessment of the rate of progression of liver fibrosis constitutes a vital aspect of this ongoing clinical investigation.

The clinical use of antibiotics in PSC is still controversial, not only considering its effectiveness, but also wary of its side effects and application time, and there is a risk of recurrence of the disease. Due to its characteristics, it may mainly target patients with severe cholangitis.

## 7 Immune modulators

Although the precise etiology of PSC is still unknown, numerous studies have demonstrated the involvement of genetic and immunological variables in the pathogenesis. Prevailing academic consensus posits that this disease is immune-mediated and genetically predisposed, thereby implicating immunosuppressive medications as potential therapeutic interventions. Examples of such agents encompass prednisolone, budesonide, azathioprine, cyclosporine, methotrexate, meclofenamic acid esters, and tacrolimus. However, extant researches indicate a notable inadequacy in the clinical efficacy of these immune modulators for PSC (36). According to the latest European guidelines, immunosuppressants should not be used as a routine therapeutic agent for PSC. A new drug, Vidofludimus calcium (IMU-838), has emerged. IMU-838 is a DHODH (dihydrolactate dehydrogenase) inhibitor that induces apoptosis of proliferating lymphocytes, resulting in decreased IL-17 and INF-γ. IMU-838 has successfully undergone a phase II trial focused on its potential for the treatment of PSC (NCT03722576). The primary objective of this trial was the improvement of ALP, with secondary endpoints encompassing the evaluation of additional liver biomarkers (bilirubin, AST, and ALT). Encouragingly, 27.7% of participants achieved ALP normalization by week 24, with no major drug-related side events (37). These results provided impetus for further research of IMU-838 as a potential therapeutic agent in PSC. A systematic review and meta-analysis associated with the effectiveness of immunomodulatory therapies in PSC revealed that immunomodulators significantly reduced ALP levels. However, normalization of ALP levels was not attained. Moreover, there was no significant change in AST levels or total bilirubin levels in PSC patients after treatment (38). The future endeavors are anticipated to involve additional randomized controlled studies with adequate sample sizes, various dosage regimens, longer treatment durations. Such endeavors aim to provide a comprehensive evaluation of the effectiveness of potential immunomodulatory interventions in the context of PSC, thereby contributing to the refinement of therapeutic strategies for this intricate hepatobiliary disorder. However, immunomodulators are generally expensive and not suitable for patients with poor economic conditions.

## 8 Steroid hormones

It is expected that budesonide, a corticosteroid with a high affinity for receptors connected to hepatic metabolism, will be effective in PSC due to its established effectiveness in disease with immunologic effects. In a prospective, non-randomized trial examining the effectiveness of budesonide (9 mg/d) in PSC, it is found that the ALP and AST were significantly improved at the 1-year mark. However, no significant effect on the degree of fibrosis or disease stage was observed. And side effects, including bone loss in the femoral neck and lumbar spine, were recorded (39). In a brief investigation involving 12 patients, prednisone (10 mg/d) and colchicine (0.6 mg/d bid) were used. After 2 years of treatment, no differences in liver histology or liver enzymes were identified when compared to the control group (40). In addition, glucocorticoids combined with immunosuppressants and sometimes UDCA can be used for the treatment of overlap syndrome (OS). OS refers to the coexistence of multiple liver disease features in the same patient, such as primary autoimmune hepatitis, PSC, or PBC (41). It can be seen that the efficacy of steroid hormones in PSC patients is not clear, and further research is needed. Moreover, hormones have certain side effects, especially pay attention to their application in patients with diabetes and other autoimmune diseases.

## 9 Other biologicals

### 9.1 Cenicriviroc

C-C chemokine receptor types 2 and 5 (CCR2/CCR5) are promising therapeutic targets of PSC due to their involvement in fibrosis and inflammation. Cenicriviroc (TAK-652, CVC) is a new dual inhibitor targeting both CCR2 and CCR5. In order to elucidate the safety and efficacy of CVC, an exploratory phase II clinical trial characterized by a single-arm and open-label design was conducted at eight sites in the United States and Canada. The entry criteria were set as follow: PSC patients with or without inflammatory bowel illness, exhibiting levels equal to or exceeding 1.5 times the upper limit of normal. Following a 24-week course of CVC treatment, the most frequent side effects were rash, fatigue, and dizziness. Notably, these side effects were reported at varying frequencies. Importantly, the trial findings indicated that CVC was generally well-tolerated among the studies (42) (NCT02653625).

### 9.2 CM-101

Extaxin-2, also known as C-C chemokine ligand 24 (CCL24), is a common chemotactic cytokine implicated in the development of pulmonary fibrosis. CCL24 and its receptor have also been shown to stimulate collagen synthesis. The monoclonal antibody CM-101, targeting CCL24, has exhibited efficacy in the reduction of inflammation and fiber pathways in many diseases (43, 44). A current phase II randomized, double-blind, placebo-controlled clinical trial is underway to assess the efficacy and safety of CM-101 in PSC (NCT04595825). Furthermore, a investigation in Mdr2−⁣/⁣− mice has demonstrated that the CM-101 administration significantly reduces inflammation, fibrosis, and cholestasis-associated markers in the biliary area (45). There is also a phase II multicenter 12-week study (EUCTR2018-004258-77-GB) assessing the safety, effectiveness, and tolerability of CM-101 in patients with PSC. However, enrollment for this trial has not yet started.

### 9.3 Vedolizumab

Individuals with inflammatory bowel disease (IBD) are treated with vedolizumab, a biological agent that inhibits α4β7 integrin and encourages lymphocyte infiltration into the colon. However, there is little clinical data on its efficacy in PSC treatment. A study systematically collected data from patients with PSC and IBD, who had received at least three doses of vedolizumab. The study, conducted across European and North American centers of the International PSC Study Group, examined the overall changes in patients’ liver biochemical characteristics as well as the proportion of patients who had significant reduction ALP levels (20% or more). It is found that only patients with cirrhosis had a 20% or more decline in ALP levels, 21 patients experienced liver-related complications. No evidence of a biochemical response to vedolizumab was identified in this worldwide study group’s investigation (46). The administration of vedolizumab did not result in any improvement in liver biochemistry among pediatric patients with PSC-IBD, as indicated by a study examining hepatic outcomes in this population (47). These results indicate that vedolizumab has not shown positive results in improving liver biochemical markers in patients with PSC-IBD, and its efficacy remains in IBD.

### 9.4 HTD1801

Orally administered lipid-lowering medication HTD1801 (berberine ursodeoxycholate, BUDCA), an ionic salt of berberine and UDCA, manifests a diverse spectrum of metabolic activities. According to earlier research, HTD1801 shows a significant metabolic effect in non-alcoholic steatohepatitis (NASH) and diabetes (NCT03656744) (48). Additionally, Kowdley et al. (48) carried out an 18-week research in which 55 PSC patients were randomized to receive placebo (*n* = 16), HTD1801 500 mg bid (*n* = 15), or HTD1801 1,000 mg bid (*n* = 24). This study was utilized to establish the efficacy and safety of HTD1801 in PSC. After 6 weeks, there was a considerable decrease in ALP level in the HTD1801 1,000 mg dose group. Importantly, this reduction in ALP levels was accompanied by favorable tolerability of the medication. These findings collectively suggest that HTD1801 holds promise in ameliorating ALP levels and may emerge as a potential therapeutic avenue for individuals grappling with PSC (NCT03333928) (49).

### 9.5 Bexotegrast

It has been demonstrated in preclinical investigations that bexotegrast (PLN-74809), a potent inhibitor of the αvβ6/αvβ1 dual integrins, is a highly efficacious agent for preventing integrin-mediated activation of transforming growth factor-β induced fibrosis in pulmonary fibrosis (50). In order to assess the safety, tolerability, and pharmacokinetics of PLN-74809 in PSC, a randomized, double-blind, placebo-controlled phase IIa trial is actively enrolling participants (NCT04480840).

### 9.6 HK-66oS

Through raising intracellular nicotinamide adenine dinucleotide (NAD+) levels, HK-66oS (Beta-Lapachone), a potent NAD+ enhancer, exhibits notable anti-inflammatory and anti-fibrotic properties. To assess the safety and effectiveness of HK-66oS in PSC, an ongoing phase II clinical trial, designed as a randomized, double-blind, and placebo-controlled study with a duration of 12 weeks, is actively recruiting participant (KCT0006590). This clinical investigation aims to provide rigorous scientific insights into the therapeutic potential of HK-66oS in the context of PSC.

## 10 Stem cells

Mesenchymal stem cells (MSCs) are distinguished by their capacity for multilineage differentiation potential and rendering them the most common cell source for stem cell therapy. MSCs play an important role in tissue regeneration and immune response regulation. It has been verified that human placenta-derived MSC can alleviate PSC in mouse models and organoid models by regulating TGR5, thereby ameliorating cholestasis and liver inflammation (51). The mechanism of action of stem cell therapy involves immune regulation, anti-fibrosis, and tissue repair (3). Presently, a phase I, characterized by a randomized controlled design and an open approach, is underway to evaluate the safety and tolerability of umbilical cord MSC injection in healthy subjects (NCT03516006). This investigation seeks to contribute valuable insights into the safety profile and feasibility of employing umbilical cord-derived MSCs, thereby advancing our understanding of their potential therapeutic applications. However, due to the limited progress of current research, uncertain prospects, and costly clinical applications, the time of realization is unknown.

The application of these biologics in PSC is still in the preliminary research stage, but some have shown some effect in other diseases. For example, HTD1801 is a lipid-lowering agent, PLN-74809 can block pulmonary fibrosis, and we can think flexibly in the future, and recommend other drugs with similar mechanisms of action to continuously explore new possibilities.

## 11 Conclusion

Based on the clinical trials included in this article, currently, there are many studies that try to provide more treatment options for blocking and reversing PSC progression from various potential mechanisms of PSC. Developing different drugs for different individuals with the purpose of personalized treatment. With the exploration of the pathogenesis of PSC, therapeutic agents are surfacing and progressing into clinical trials. However, the pathological mechanisms of PSC not only involve immune-mediated inflammation but also include non-immune factors such as biliary tract injury, fibrosis, and bile acid metabolism disorders. These drugs cannot comprehensively cover the complex pathological processes of PSC, which may be one of the reasons for the poor efficacy of traditional drugs. Newer immunomodulatory agents (such as IMU-838) have shown a broader prospect in the treatment of PSC compared to traditional corticosteroids and classic immunosuppressive agents. Their selective immunomodulatory mechanisms can more precisely target the pathological processes of PSC. However, these drugs are still in the clinical trial phase and require further long-term studies to verify their actual efficacy and safety in the treatment of PSC. Novel modalities such as stem cell therapies to complement established pharmaceutical interventions. Indeed, there are still a series of unresolved treatment issues in PSC. For example, the recommended drugs, such as UDCA, have difficulty in blocking the progression of the disease. Additionally, there is a lack of effective drugs based on immunological treatment. Furthermore, the relatively low incidence of PSC makes clinical research challenging. Therefore, as a difficult-to-treat disease, the clinical management of PSC remains challenging. ALP is easy to detect and can be dynamically monitored, so it is often used as an endpoint indicator. However, solely relying on ALP to measure disease progression has its limitations, its correlation with long-term outcomes (such as the need for liver transplantation, liver failure or survival rate) is not yet fully clear, and it cannot be used alone as a reliable indicator for evaluating disease prognosis. In clinical practice, it is necessary to combine other indicators and clinical symptoms to assess disease outcomes. Currently, in addition to ALP, the main alternative endpoint indicators include liver biopsy, liver stiffness measurement, magnetic resonance cholangiopancreatography, and some prediction models, such as UK-PSC. These indicators provide a more comprehensive assessment tool for the clinic, aiding in individualized treatment (3). These may provide a more sensitive measure of treatment response. However, it is hopeful that the aforementioned candidate drugs may bring more treatment options for PSC, and the future prospects are promising. However, it is necessary to include fibrosis and long-term clinical prognosis indicators for a more comprehensive evaluation in the future. The ideal strategy for treating PSC management may entail the combination of multiple drugs exhibiting distinct mechanisms of action. According to the above clinical trials, combination therapy is a good choice, especially in improving biochemical indicators. However, the research on combination therapy is still in the early stage, due to the combined use of multiple medications, side effects will also be additive, and the appropriate population needs to be carefully assessed, and its long-term efficacy and safety need to be further verified. Future research needs to combine more clinical data to optimize the combination treatment program and provide more effective treatment options for PSC patients.

## Patient and public involvement

Patients and/or the public were not involved in the design, or conduct, or reporting, or dissemination plans of this research.

## References

[1] European Association for the Study of the Liver. EASL Clinical Practice Guidelines on sclerosing cholangitis. *J Hepatol.* (2022) 77:761–806. 10.1016/j.jhep.2022.05.011 35738507

[2] Lillemoe K Pitt H Cameron J. Primary sclerosing cholangitis. *Surg Clin North Am*. (1990) 70:1381–402. 10.1016/s0039-6109(16)45290-4 2247821

[3] Assis D Bowlus C. Recent advances in the management of primary sclerosing cholangitis. *Clin Gastroenterol Hepatol*. (2023) 21:2065–75. 10.1016/j.cgh.2023.04.004 37084929

[4] van Munster K Bergquist A Ponsioen C. Inflammatory bowel disease and primary sclerosing cholangitis: One disease or two? *J Hepatol*. (2024) 80:155–68. 10.1016/j.jhep.2023.09.031 37940453

[5] Trivedi P Hirschfield G Adams D Vierling J. Immunopathogenesis of primary biliary cholangitis, primary sclerosing cholangitis and autoimmune hepatitis: Themes and concepts. *Gastroenterology*. (2024) 166:995–1019. 10.1053/j.gastro.2024.01.049 38342195

[6] Beuers U Trauner M Jansen P Poupon R. New paradigms in the treatment of hepatic cholestasis: From UDCA to FXR, PXR and beyond. *J Hepatol.* (2015) 62(1 Suppl):S25–37. 10.1016/j.jhep.2015.02.023 25920087

[7] Lindor K. Ursodiol for primary sclerosing cholangitis. Mayo primary sclerosing cholangitis-ursodeoxycholic acid study group. *N Engl J Med*. (1997) 336:691–5. 10.1056/NEJM199703063361003 9041099

[8] Olsson R Boberg K de Muckadell O Lindgren S Hultcrantz R Folvik G High-dose ursodeoxycholic acid in primary sclerosing cholangitis: A 5-year multicenter, randomized, controlled study. *Gastroenterology*. (2005) 129:1464–72. 10.1053/j.gastro.2005.08.017 16285948

[9] Lindor K Kowdley K Luketic V Harrison M McCashland T Befeler A High-dose ursodeoxycholic acid for the treatment of primary sclerosing cholangitis. *Hepatology*. (2009) 50:808–14. 10.1002/hep.23082 19585548 PMC2758780

[10] Eaton J Silveira M Pardi D Sinakos E Kowdley K Luketic V High-dose ursodeoxycholic acid is associated with the development of colorectal neoplasia in patients with ulcerative colitis and primary sclerosing cholangitis. *Am J Gastroenterol*. (2011) 106:1638–45. 10.1038/ajg.2011.156 21556038 PMC3168684

[11] Triantos C Koukias N Nikolopoulou V Burroughs A. Meta-analysis: Ursodeoxycholic acid for primary sclerosing cholangitis. *Aliment. Pharmacol. Ther*. (2011) 34:901–10. 10.1111/j.1365-2036.2011.04822.x 21883323

[12] Trauner M Fuchs C Halilbasic E Paumgartner G. New therapeutic concepts in bile acid transport and signaling for management of cholestasis. *Hepatology*. (2017) 65:1393–404. 10.1002/hep.28991 27997980

[13] Fickert P Wagner M Marschall H Fuchsbichler A Zollner G Tsybrovskyy O 24-norUrsodeoxycholic acid is superior to ursodeoxycholic acid in the treatment of sclerosing cholangitis in Mdr2 (Abcb4) knockout mice. *Gastroenterology*. (2006) 130:465–81. 10.1053/j.gastro.2005.10.018 16472600

[14] Fickert P Hirschfield G Denk G Marschall H Altorjay I Färkkilä M norUrsodeoxycholic acid improves cholestasis in primary sclerosing cholangitis. *J Hepatol*. (2017) 67:549–58. 10.1016/j.jhep.2017.05.009 28529147

[15] Gerussi A Lucà M Cristoferi L Ronca V Mancuso C Milani C New therapeutic targets in autoimmune cholangiopathies. *Front Med*. (2020) 7:117. 10.3389/fmed.2020.00117 32318580 PMC7154090

[16] Kowdley K Vuppalanchi R Levy C Floreani A Andreone P LaRusso N A randomized, placebo-controlled, phase II study of obeticholic acid for primary sclerosing cholangitis. *J Hepatol*. (2020) 73 94–101. 10.1016/j.jhep.2020.02.033 32165251 PMC8157171

[17] Trauner M Gulamhusein A Hameed B Caldwell S Shiffman M Landis C The nonsteroidal farnesoid X receptor agonist cilofexor (GS-9674) improves markers of cholestasis and liver injury in patients with primary sclerosing cholangitis. *Hepatology*. (2019) 70:788–801. 10.1002/hep.30509 30661255 PMC6767458

[18] Sanyal A Ling L Beuers U DePaoli A Lieu H Harrison S Potent suppression of hydrophobic bile acids by aldafermin, an FGF19 analogue, across metabolic and cholestatic liver diseases. *JHEP Rep*. (2021) 3:100255. 10.1016/j.jhepr.2021.100255 33898959 PMC8056274

[19] Hirschfield G Chazouillères O Drenth J Thorburn D Harrison S Landis C Effect of NGM282, an FGF19 analogue, in primary sclerosing cholangitis: A multicenter, randomized, double-blind, placebo-controlled phase II trial. *J Hepatol*. (2019) 70:483–93. 10.1016/j.jhep.2018.10.035 30414864

[20] Assis D Abdelghany O Cai S Gossard A Eaton J Keach J Combination therapy of all-trans retinoic acid with ursodeoxycholic acid in patients with primary sclerosing cholangitis: A human pilot study. *J Clin Gastroenterol*. (2017) 51:e11–6. 10.1097/MCG.0000000000000591 27428727 PMC5218875

[21] Mizuno S Hirano K Tada M Yamamoto K Yashima Y Yagioka H Bezafibrate for the treatment of primary sclerosing cholangitis. *J Gastroenterol*. (2010) 45:758–62. 10.1007/s00535-010-0204-x 20127368

[22] Lemoinne S Pares A Reig A Ben Belkacem K Kemgang Fankem A Gaouar F Primary sclerosing cholangitis response to the combination of fibrates with ursodeoxycholic acid: French-spanish experience. *Clin Res Hepatol Gastroenterol*. (2018) 42:521–8. 10.1016/j.clinre.2018.06.009 30100231

[23] Ghonem N Auclair A Hemme C Gallucci G de la Rosa Rodriguez R Boyer J Fenofibrate improves liver function and reduces the toxicity of the bile acid pool in patients with primary biliary cholangitis and primary sclerosing cholangitis who are partial responders to ursodiol. *Clin Pharmacol Ther*. (2020) 108:1213–23. 10.1002/cpt.1930 32480421 PMC7886378

[24] Hatami B Mosala M Hassani A Ardakani M Gholami S Zali M. Fenofibrate in primary sclerosing cholangitis; A randomized, double-blind, placebo-controlled trial. *Pharmacol Res Perspect*. (2022) 10:e00984. 10.1002/prp2.984 35822553 PMC9277608

[25] van den Hoek A Verschuren L Caspers M Worms N Menke A Princen H. Beneficial effects of elafibranor on NASH in E3L.CETP mice and differences between mice and men. *Sci Rep*. (2021) 11:5050. 10.1038/s41598-021-83974-8 33658534 PMC7930243

[26] Schattenberg J Pares A Kowdley K Heneghan M Caldwell S Pratt D A randomized placebo-controlled trial of elafibranor in patients with primary biliary cholangitis and incomplete response to UDCA. *J Hepatol*. (2021) 74:1344–54. 10.1016/j.jhep.2021.01.013 33484775

[27] Haczeyni F Wang H Barn V Mridha A Yeh M Haigh W The selective peroxisome proliferator-activated receptor-delta agonist seladelpar reverses nonalcoholic steatohepatitis pathology by abrogating lipotoxicity in diabetic obese mice. *Hepatol Commun*. (2017) 1:663–74. 10.1002/hep4.1072 29404484 PMC5721439

[28] Hegade V Jones D Hirschfield G. Apical sodium-dependent transporter inhibitors in primary biliary cholangitis and primary sclerosing cholangitis. *Dig Dis*. (2017) 35:267–74. 10.1159/000450988 28249258

[29] Bowlus C Eksteen B Cheung A Thorburn D Moylan C Pockros P Safety, tolerability, and efficacy of maralixibat in adults with primary sclerosing cholangitis: Open-label pilot study. *Hepatol Commun*. (2023) 7:e0153. 10.1097/HC9.0000000000000153 37184523 PMC10187837

[30] Caballero-Camino F Rodrigues P Wångsell F Agirre-Lizaso A Olaizola P Izquierdo-Sanchez L A3907, a systemic ASBT inhibitor, improves cholestasis in mice by multiorgan activity and shows translational relevance to humans. *Hepatology*. (2023) 78:709–26. 10.1097/HEP.0000000000000376 36999529 PMC10442107

[31] Färkkilä M Karvonen A Nurmi H Nuutinen H Taavitsainen M Pikkarainen P Metronidazole and ursodeoxycholic acid for primary sclerosing cholangitis: A randomized placebo-controlled trial. *Hepatology*. (2004) 40:1379–86. 10.1002/hep.20457 15565569

[32] Tabibian J Weeding E Jorgensen R Petz J Keach J Talwalkar J Randomised clinical trial: Vancomycin or metronidazole in patients with primary sclerosing cholangitis - a pilot study. *Aliment Pharmacol Ther*. (2013) 37:604–12. 10.1111/apt.12232 23384404

[33] Rahimpour S Nasiri-Toosi M Khalili H Ebrahimi-Daryani N Nouri-Taromlou M Azizi Z. A triple blinded, randomized, placebo-controlled clinical trial to evaluate the efficacy and safety of oral vancomycin in primary sclerosing cholangitis: A pilot study. *J Gastrointestin Liver Dis*. (2016) 25:457–64. 10.15403/jgld.2014.1121.254.rah 27981301

[34] Davies Y Cox K Abdullah B Safta A Terry A Cox K. Long-term treatment of primary sclerosing cholangitis in children with oral vancomycin: An immunomodulating antibiotic. *J Pediatr Gastroenterol Nutr*. (2008) 47:61–7. 10.1097/MPG.0b013e31816fee95 18607270

[35] Wilson H Khokhar F Enoch D Brown N Ahluwalia J Dougan G Point-prevalence survey of carbapenemase-producing *Enterobacteriaceae* and vancomycin-resistant enterococci in adult inpatients in a university teaching hospital in the UK. *J Hosp Infect*. (2018) 100:35–9. 10.1016/j.jhin.2018.06.024 29969691

[36] Lindor K Kowdley K Harrison M American College of Gastroenterology. ACG clinical guideline: Primary sclerosing cholangitis. *Am J Gastroenterol.* (2015) 110:646-59; quiz 660. 10.1038/ajg.2015.112 25869391

[37] Carey E Eaton J Clayton M Gossard A Iqbal S Ullah H A pilot study of vidofludimus calcium for treatment of primary sclerosing cholangitis. *Hepatol Commun*. (2022) 6:1589–97. 10.1002/hep4.1926 35238498 PMC9234677

[38] Liu X Wang H Liu X Bridle K Crawford D Liang X. Efficacy and safety of immune-modulating therapy for primary sclerosing cholangitis: A systematic review and meta-analysis. *Pharmacol Ther*. (2022) 237:108163. 10.1016/j.pharmthera.2022.108163 35271884

[39] Angulo P Batts K Jorgensen R LaRusso N Lindor K. Oral budesonide in the treatment of primary sclerosing cholangitis. *Am J Gastroenterol*. (2000) 95:2333–7. 10.1111/j.1572-0241.2000.02323.x 11007238

[40] Lindor K Wiesner R Colwell L Steiner B Beaver S LaRusso N. The combination of prednisone and colchicine in patients with primary sclerosing cholangitis. *Am J Gastroenterol.* (1991) 86:57–61. 1986556

[41] Guifarro D De Oliveira-Gomes D Beas R Yibirin-Wakim M Montalvan-Sanchez E. Primary sclerosing cholangitis-autoimmune hepatitis overlap syndrome: Significant barriers in liver disease diagnosis and treatment experienced by the latino community. *Cureus*. (2023) 15:e36126. 10.7759/cureus.36126 37065404 PMC10099648

[42] Eksteen B Bowlus C Montano-Loza A Lefebvre E Fischer L Vig P Efficacy and safety of cenicriviroc in patients with primary sclerosing cholangitis: Perseus study. *Hepatol Commun*. (2020) 5:478–90. 10.1002/hep4.1619 33681680 PMC7917279

[43] Segal-Salto M Barashi N Katav A Edelshtein V Aharon A Hashmueli S A blocking monoclonal antibody to CCL24 alleviates liver fibrosis and inflammation in experimental models of liver damage. *JHEP Rep*. (2020) 2:100064. 10.1016/j.jhepr.2019.100064 32039405 PMC7005554

[44] Barashi N Segal M Aharon A Katav A Mor A Mishalian I CCL24 modulates fibrosis development in primary sclerosing cholangitis: Correlation of human serum CCL24 levels with fibrosis markers and data from the Mdr2–/– mouse model. *J Hepatol*. (2020) 73:S486. 10.1016/S0168-8278(20)31452-5

[45] Greenman R Segal-Salto M Barashi N Hay O Katav A Levi O CCL24 regulates biliary inflammation and fibrosis in primary sclerosing cholangitis. *JCI Insight*. (2023) 8:e162270. 10.1172/jci.insight.162270 37345655 PMC10371243

[46] Lynch K Chapman R Keshav S Montano-Loza A Mason A Kremer A Effects of vedolizumab in patients with primary sclerosing cholangitis and inflammatory bowel diseases. *Clin Gastroenterol Hepatol.* (2020) 18:179–87.e6. 10.1016/j.cgh.2019.05.013 31100458 PMC6941216

[47] Laborda T Ricciuto A Aumar M Carman N DiGuglielmo M Draijer L Vedolizumab therapy in children with primary sclerosing cholangitis: Data from the pediatric primary sclerosing cholangitis consortium. *J Pediatr Gastroenterol Nutr*. (2020) 71:459–64. 10.1097/MPG.0000000000002855 32740528

[48] Kowdley K Forman L Eksteen B Gunn N Sundaram V Landis C A randomized, dose-finding, proof-of-concept study of berberine ursodeoxycholate in patients with primary sclerosing cholangitis. *Am J Gastroenterol*. (2022) 117:1805–15. 10.14309/ajg.0000000000001956 36327436

[49] Harrison S Gunn N Neff G Kohli A Liu L Flyer A A phase 2, proof of concept, randomised controlled trial of berberine ursodeoxycholate in patients with presumed non-alcoholic steatohepatitis and type 2 diabetes. *Nat Commun*. (2021) 12:5503. 10.1038/s41467-021-25701-5 34535644 PMC8448729

[50] Decaris M Schaub J Chen C Cha J Lee G Rexhepaj M Dual inhibition of αvβ6 and αvβ1 reduces fibrogenesis in lung tissue explants from patients with IPF. *Respir Res*. (2021) 22:265. 10.1186/s12931-021-01863-0 34666752 PMC8524858

[51] Yao Q Chen W Yu Y Gao F Zhou J Wu J Human placental mesenchymal stem cells relieve primary sclerosing cholangitis via upregulation of TGR5 in Mdr2-/- mice and human intrahepatic cholangiocyte organoid models. *Research*. (2023) 6:0207. 10.34133/research.0207 37600495 PMC10433880

